# Efficient pretreatment of lignocellulosic biomass with high recovery of solid lignin and fermentable sugars using Fenton reaction in a mixed solvent

**DOI:** 10.1186/s13068-018-1288-4

**Published:** 2018-10-20

**Authors:** Hui-Tse Yu, Bo-Yu Chen, Bing-Yi Li, Mei-Chun Tseng, Chien-Chung Han, Shin-Guang Shyu

**Affiliations:** 1 Institute of Chemistry, Academia Sinica, Taipei, 11529 Taiwan; 2 Department of Chemistry, National Tsing Hua University, Hsinchu, 30013 Taiwan

**Keywords:** Pretreatment, Fenton reaction, Corncob, Xylose, Lignin

## Abstract

**Background:**

Pretreatment of biomass to maximize the recovery of fermentable sugars as well as to minimize the amount of enzyme inhibitors formed during the pretreatment is a challenge in biofuel process. We develop a modified Fenton pretreatment in a mixed solvent (water/DMSO) to combine the advantages of organosolv and Fenton pretreatments. The hemicellulose and cellulose in corncob were effectively degraded into xylose, glucose, and soluble glucose oligomers in a few hours. This saccharide solution, separated from the solid lignin simply by filtration, can be directly applied to the subsequent enzymatic hydrolysis and ethanol fermentation.

**Results:**

After the pretreatment, 94% carbohydrates were recovered as soluble monosaccharide (xylose and glucose) and glucose oligomers in the filtrates, and 87% of solid lignin was recovered as the filter residue. The filtrates were directly applied to enzymatic hydrolysis, and 92% of raw corncob glucose was recovered. The hydrolysates containing the glucose and xylose from the enzymatic hydrolysis were directly applied to ethanol fermentation with ethanol yield equals 79% of theoretical yield. The pretreatment conditions (130 °C, 1.5 bar; 30 min to 4 h) are mild, and the pretreatment reagents (H_2_O_2_, FeCl_3_, and solvent) had low impact to environment. Using ferrimagnetic Fe_3_O_4_ resulted in similar pretreatment efficiency and Fe_3_O_4_ could be removed by filtration.

**Conclusions:**

A modified Fenton pretreatment of corncob in DMSO/water was developed. Up to 94% of the carbohydrate content of corncob was recovered as a saccharide solution simply by filtration. Such filtrate was directly applied to the subsequent enzymatic hydrolysis and where 92% of the corncob glucose content was obtained. The hydrolysate so obtained was directly applied to ethanol fermentation with good fermentability. The pretreatment method is simple, and the additives and solvents used have a low impact to the environment. This method provides the opportunity to substantially maximize the carbohydrate and solid lignin recovery of biomass with a comparatively green process, such that the efficiency of biorefinery as well as the bioethanol production process can be improved. The pretreatment is still relatively energy intensive and expensive, and further optimization of the process is required in large-scale operation.

**Electronic supplementary material:**

The online version of this article (10.1186/s13068-018-1288-4) contains supplementary material, which is available to authorized users.

## Background

Fermentation of sugar from biomass to ethanol is one of the most important bioenergy technologies [1–3]. Biomass, a lignocellulosic material, has three major components: lignin, cellulose, and hemicellulose [4]. Hemicellulose and cellulose are the feedstock of fermentable sugar. Among many methods to hydrolyze these polysaccharides to fermentable sugar, enzymatic hydrolysis is the most commonly used method in the present bioethanol industry [5, 6].

Enzymatic hydrolysis (cellulose saccharification) cannot be directly applied to biomass, because cellulose in biomass is protected by lignin and hemicellulose [7]. Thus, pretreatment of biomass is needed to enhance the accessibility of enzymes to cellulose, to maximize the recovery of cellulose, and, at the same time, to minimize the amount of enzyme inhibitors that form during the pretreatment process [8].

Recently, enlightened by the white-rot or brown-rot fungi lignin degradation via in vivo Fenton chemistry [9], Fenton oxidation was applied to the pretreatment of lignocellulosic biomass [10–18] and degradation of cellulose [19, 20]. Combination of Fenton oxidation with the other pretreatment methods was reported to improve the pretreatment, so that subsequent enzymatic hydrolysis and fermentation steps could be optimized [15–18]. Despite the mild reaction conditions (low pressure and temperature) [10–20], environmentally benign reagents [21] and improved cellulose/lignin ratio of the pretreated biomass [16], a non-negligible amount of cellulose (up to 65% of glucan), sugar content (up to 60% of carbohydrate), and lignin (up to 60%) of the raw biomass were lost after Fenton pretreatment [13, 18].

A detailed molecular mechanism underlying the Fenton pretreatment process has yet to be described. It is generally accepted that the HO**·** (hydroxyl) and HOO**·** (perhydroxyl) radicals generated by the Fenton reaction degrade the lignocellulosic structure [10]. To improve the efficiency of the Fenton pretreatment, it is critical to enhance the accessibility of these free radicals to the lignocellulosic structure. Organosolv pretreatment allows the penetration of the pretreatment solution (organic solvent) into the lignocellulosic structure and leads to separation of high-purity cellulose by dissolving most lignin and hemicellulose [8]. Adding organic solvent into the Fenton pretreatment may help to dissolve some of the lignin and hemicellulose. This would then allow the free radicals from the Fenton reaction to penetrate deeper into the interior framework of the lignocellulosic structure. The aim of the present study is to develop a modified Fenton pretreatment in a mixed solvent (water and organic solvent) system which can maximize the carbohydrate and lignin recovery of the biomass.

We choose corncob as our pretreatment substrate as corn is grown on scale and large amounts of agricultural waste (corn residue) are generated. Maize residue is also one of the most abundant raw materials for biocompatible products along with bagasse, rice straw, wheat straw, and other lignocellulose substrates [22]. Corncob, a by-product of the sweet corn processing industry, is available in sufficient quantity. Pretreatments of corncob for biofuels have been of increasing interest in the recent years [23–33], because corncob contains a larger portion of glucose and xylose. In addition to maximize the glucose recovery, an efficient pretreatment method for corncob should provide high xylose yield, because xylose has many broad applications [34, 35].

Herein, we report a modified Fenton pretreatment which combines the advantage of organosolv and Fenton pretreatments. The hemicellulose and cellulose in corncob were effectively degraded into xylose, glucose, and soluble glucose oligomers in the pretreatment, and the saccharide solution was separated from the solid lignin simply by filtration. Up to 94% of the carbohydrate content of corncob was recovered, and the saccharide solution was directly applied to the subsequent hydrolysis by cellulase. Up to 92% of the glucose content of the corncob was recovered. The time of hydrolysis as well as the amount of cellulase needed for digestion were greatly reduced. Furthermore, the hydrolysates containing the glucose and xylose from the enzymatic hydrolysis can be directly applied to ethanol fermentation.

## Materials and methods

### Lignocellulose biomass and chemicals

Corncobs were obtained domestically (Tainan, Taiwan) and were washed with deionized water. After drying at 105 °C, corncobs were mechanically grinded into particles and sieved through 40 mesh sieves (particle size smaller than 0.49 mm). All chemical reagents were purchased from commercial sources and used without further purification. Iron (III) chloride, *o*-phenanthroline, and dimethyl sulfoxide (DMSO) were purchased from Aldrich and J.T. Baker, respectively. Glucose and gluconic acid were purchased from Alfa Aesar. Hydrogen peroxide solution (35 wt% in H_2_O), α-cellulose, and cellulase from *Trichoderma reesei* were purchased from Sigma-Aldrich. *S. cerevisiae* for fermentation was purchased from Algist Bruggeman.

### Biomass composition and characterization

The composition of the corncob particles was determined by following the standard protocol of the National Renewable Energy Laboratory [36]. The amount of xylose, glucose, and arabinose were determined by high-performance liquid chromatography (HPLC) on a Waters (1525 pump) with a 25 cm × 4.6 mm Shodex Asahipak NH2P-50 4E column using acetonitrile/water (4:1) as an eluent at a flow rate of 1.0 mL/min at 35 °C or with a 25 cm × 4.6 mm Benson BP-800H^+^ column using 5.0 mM H_2_SO_4_ aqueous solution as an eluent at a flow rate of 0.5 mL/min at 85 °C. The quantification of HMF, furfural, and gluconic acid were performed by Bruker Advance UHPLC system coupled to a Bruker EVOQ EliteTM triple quadrupole mass (Bremen, Germany) equipped with an atmospheric pressure chemical ionization (APCI) and electrospray (ESI) interfaces [37]. Chromatographic separations were performed on a Waters Acquity UPLC BEH C18 column (2.1 × 100 mm, 1.7 μm) using an isocratic mixture of 0.01 mmol/L acetic acid in 0.2% aqueous solution of formic acid for HMF and furfural, and on a Merck ZIC-HILIC column (2.1 × 150 mm, 3.5 μm) using mobile phase A (acetonitrile modified with 0.1% (v/v) formic acid) and mobile phase B (5.0 mmol/L ammonium acetate modified with 0.1% (v/v) formic acid) with gradient profile 10% B to 90% B in 19 min for glucose and gluconic acid. Both analyses were performed at a flow rate of 0.30 mL/min. The total carbohydrates content was determined by the phenol–sulfuric acid method [38]. Mineral contents were determined by following the standard protocol of the National Renewable Energy Laboratory [36].

### Pretreatment method

The pretreatment reagent solution was prepared by dissolving FeCl_3_ (7.5 × 10^−3^ mmol) and H_2_O_2_ (0.30 mmol, 0.26 mL, 35 wt% in H_2_O) in the solvent (2.0 mL, DMSO/H_2_O = 1:6) in a Pyrex tube with a Teflon screw cap. The solution was then stirred at 130 °C for 10–15 min before use.

Corncob powder (0.200 g, particle size smaller than 0.49 mm) was added into the reagent solution and stirred at 130 °C for 30 min in a Pyrex tube with a Teflon screw cap. The slurry was then filtered, and a light brown powder and a brown filtrate were obtained. The amount of glucose, xylose, arabinose, and total carbohydrates in the filtrate were determined by quantitative HPLC and phenol–sulfuric acid method, respectively.

**Fig. 1:**
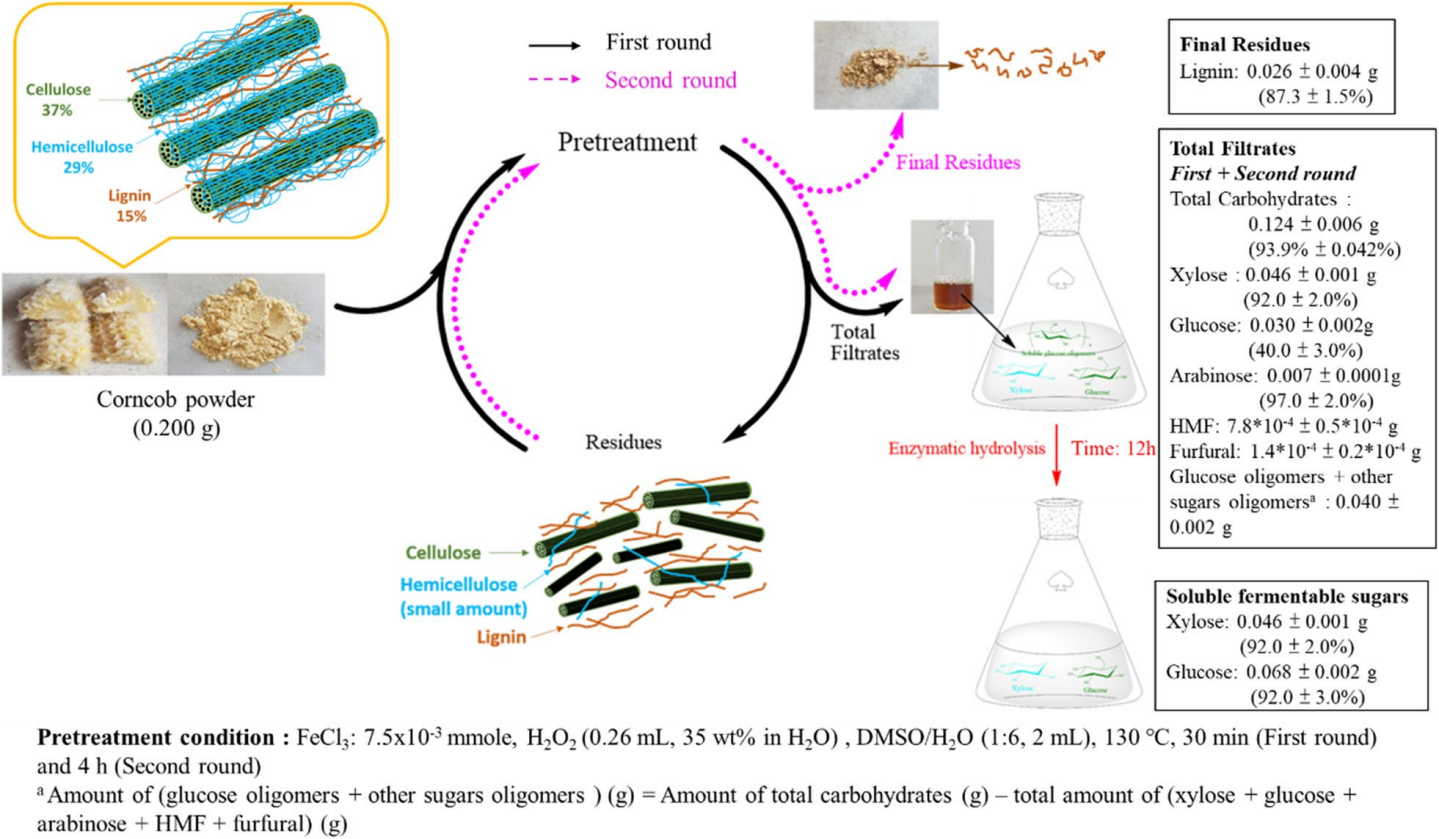
Flowchart and results of the corncob pretreatment

The light brown powder obtained in the above step (0.084 g) was added into a fresh pretreatment reagent solution, and the mixture was stirred at 130 °C for 4 h in a Pyrex tube with a Teflon screw cap. The mixture was then filtered. A light brown powder and a brown filtrate were obtained. The amount of glucose, xylose, arabinose, and total carbohydrates in the filtrate were determined by quantitative HPLC and phenol–sulfuric acid method, respectively. TGA analysis of the light brown powder (dried at 80 °C for 12 h before TGA analysis) indicates the powder contains lignin (Additional file 1: Figure. S1) [39]. The pretreatment flow chart is shown in Fig. 1.

Product yields of the pretreatment were calculated as follows:

$${\text{Xylose yield }} = \, ({{\text{The amount of xylose produced}} \mathord{\left/ {\vphantom {{\text{The amount of xylose produced}} {\text{The amount of xylose in the feedstock}}}} \right. \kern-0pt} {\text{The amount of xylose in the feedstock}}}) \times 100\%$$

$${\text{Glucose yield }} = \, \left( {{{\text{The amount of glucose produced}} \mathord{\left/ {\vphantom {{\text{The amount of glucose produced}} {\text{The amount of glucose in the feedstock}}}} \right. \kern-0pt} {\text{The amount of glucose in the feedstock}}}} \right) \times 100\%$$

$${\text{Arabinose yield }} = \, \left( {{{\text{The amount of arabinose produced}} \mathord{\left/ {\vphantom {{\text{The amount of arabinose produced}} {\text{The amount of arabinose in the feedstock}}}} \right. \kern-0pt} {\text{The amount of arabinose in the feedstock}}}} \right) \times 100\%$$

$${\text{Lignin yield }} = \, ({{\text{The amount ofligninproduced}} \mathord{\left/ {\vphantom {{\text{The amount ofligninproduced}} {\text{The amount of ligninin the feedstock}}}} \right. \kern-0pt} {\text{The amount of ligninin the feedstock}}}) \times 100\%$$

$${\text{Total carbohydrates yield }} = \, \left( {{{\text{The amount of total carbohydrates produced}} \mathord{\left/ {\vphantom {{\text{The amount of total carbohydrates produced}} {\text{The amount of total carbohydrates in the feedstock}}}} \right. \kern-0pt} {\text{The amount of total carbohydrates in the feedstock}}}} \right) \times 100\% .$$

### Quantitative Fe(II) analysis before and during pretreatment

The concentration of the Fe(II) was determined by *o*-phenanthroline-based detection technique using Agilent Technologies Cary 8454 UV–Vis spectrometer at room temperature [40]. The *ε* (molar absorptivity at 508 nm) of Fe(II)(Phen)_3_Cl_2_ in DMSO/water (1:6) is 0.048 M^−1^ cm^−1^.

### Enzymatic hydrolysis

Enzymatic hydrolysis of the glucose oligomers in the total filtrate (combination of filtrates from the first and the second rounds of the pretreatment) was carried out using cellulase from *Trichoderma reesei*. The pH of the filtrate was adjusted to 4.5–4.8 by adding 0.15 g calcium carbonate to 4.0 mL filtrate. Cellulase (44 mg) was added into the tube, and the solution was stirred at 180 rpm for 12 h at 50 °C. The enzymatic hydrolysis was terminated by boiling the reaction mixtures at 85 °C for 5 min. The amount of glucose and xylose in the ultimate enzymatic hydrolysate was determined by HPLC.

Two control groups with similar amount of glucose content of the glucose oligomer (0.038 g) in the filtrate were applied to hydrolysis in a citrate buffer (4.0 mL; 50 mmol/L, pH 4.8) with 44 mg of cellulase under the same hydrolysis condition. One control is 0.038 g of commercial cellulose. The other is 0.103 g of corncob powder. The time course of glucose yield of the enzymatic hydrolysis results is shown in Fig. 4. The time course of the total glucose yield (both glucose obtained in the enzymatic hydrolysis and the glucose produced from the pretreatment of 0.200 g corncob) in the filtrate and the glucose yield of raw corncob (0.200 g) after enzymatic hydrolysis is shown in Fig. 3.

### Fermentability of the recovered hydrolyzates

Ethanol fermentation of the hydrolyzates from the enzymatic hydrolysis of the pretreatment filtrate was conducted using *S. cerevisiae* [15]. Fermentation of the hydrolyzates (containing 11.5 g/L glucose) was performed in a rotatory shaker at 37 °C and 150 rpm for 64 h. After fermentation, the amount of ethanol was determined by GC analyses with isopropanol as an internal standard on an Agilent 6890 Gas Chromatograph equipped with DB-5MS column (30 m × 0.25 mm internal diameter and 0.25 mm film thickness) and an FID detector. The amount of glucose was determined by high-performance liquid chromatography (HPLC).

## Results and discussion

### Pretreatment general procedure and its performance

The composition of the corncob powder was determined as follows: 66.2% of total carbohydrates, 37.3% of glucose, 25.1% of xylose, 3.7% of arabinose, 14.9% of lignin, and 3.9% of ash. The corncob powder was added into the pretreatment reagent solution [Fe(II) concentration 0.38 mM; pH 2.0 at room temperature] in a screw capped Pyrex tube and the slurry was stirred for 30 min at 130 °C. During the pretreatment process, the Fe(II) raised to 2.24 mM after 30 min, and the pH value of the mixture remained similar (2.0). The reaction was monitored by a pressure gauge, and the maximum pressure observed was 1.5 bar. After filtration, a light brown solid and a brown filtrate were obtained. The filtrate was analyzed for xylose, glucose, arabinose, and total carbohydrate contents. The contents of the filtrate were determined as follows: 0.099 g of total carbohydrates, 0.014 g of glucose, 0.045 g of xylose, 0.007 g of arabinose, 0.032 g of glucose oligomer, and other sugars. The brown residue was applied to the second-round pretreatment. After filtration, solid lignin was obtained. The contents of the second filtrate were determined as follows: 0.025 g of total carbohydrates, 0.016 g of glucose, 0.001 g of xylose, 0.008 g of glucose oligomer, and other sugars. The total filtrate (combination of filtrates from the first and the second rounds) was subjected to enzymatic hydrolysis. The flowchart of a typical corncob pretreatment is shown in Fig. 1.

The total carbohydrates in the two filtrates were 0.124 g corresponding to 94% of carbohydrates in the corncob. The total xylose and glucose content (both glucose and its glucose oligomers) in the two filtrates were 0.046 g and 0.068 g, respectively. This indicates that 92% of xylose and 92% of glucose in the corncob were recovered in the filtrate. TGA analysis of the residues (Additional file 1: Figure S1) obtained in the second pretreatment cycle indicated that the residue contains 87% of lignin in the corncob.

Despite the pretreatment temperature being higher than that of the traditional Fenton pretreatments, the pretreatment condition is still relatively mild (130 °C and 1.5 bar) as compared to other pretreatments which usually require higher pressure and temperature [8, 33, 41]. In addition, the pretreatment has several unique properties: First, the concentration of FeCl_3_ is much lower (3.3 mmol/L) than that of other Fenton and metal salt pretreatments (0.02 mol/L to 0.2 mol/L) [18, 41]. Low FeCl_3_ concentration can reduce the environmental impact and the negative influence of the FeCl_3_ in the subsequent enzymatic hydrolysis [42, 43]. Second, hydrogen peroxide is a comparatively green oxidant, and the concentration of H_2_O_2_ (1.3 mol/L) is lower than that of the other reported Fenton pretreatments (1.5 mol/L to 2.5 mol/L) [13, 18]. Lower concentration of H_2_O_2_ can reduce the loss of lignin and cellulose content [18]. Finally, the amount of DMSO, a green solvent [44], used in the solvent system is only 14.3 vol%. This DMSO concentration does not inhibit enzyme activity in many enzyme processes [45], so that the hydrolysate, containing 94% of the carbohydrate content of corncob, can be directly applied to the subsequence glucose oligomers’ enzymatic hydrolysis.

### The role of metal salt and hydrogen peroxide in the pretreatment

Because corncob contains large amounts of xylose, we used the yield of xylose as a guide post to evaluate the relationship between individual components and the performance of the pretreatment. The results are summarized in Table 1.

**Table 1:** Conditions and products of the first-round corncob pretreatment

Entry	FeCl_3_/H_2_O_2_/solvent	Xylose yield (%)	Xylose (g/L)	Glucose yield (%)	Glucose (g/L)	Arabinose yield (%)	Total carbohydrates yield (%)
1	–/H_2_O_2_/DMSO + H_2_O (1:6)	6.5 ± 0.4	1.43 ± 0.05	2.9 ± 0.3	0.94 ± 0.07	2.8 ± 0.4	15.4 ± 0.7
2	FeCl_3_/–/DMSO + H_2_O (1:6)	5.2 ± 0.4	1.15 ± 0.07	4.8 ± 0.1	1.54. ± 0.05	5.8 ± 0.6	31.4 ± 0.8
3	FeCl_3_/H_2_O_2_/DMSO + H_2_O (1:6)	92.2 ± 1.8	20.42 ± 0.68	20.1 ± 0.8	6.55 ± 0.07	98.4 ± 1.1	74.4 ± 1.1
4	FeCl_3_/H_2_O_2_/DMSO + H_2_O (3:1)	66.8 ± 0.7	14.77 ± 0.15	15.2 ± 0.3	4.95 ± 0.11	64.3 ± 0.9	61.3 ± 0.9
5	FeCl_3_/H_2_O_2_/DMSO + H_2_O (1:3)	85.3 ± 1.1	18.80 ± 0.35	16.5 ± 0.6	5.40 ± 0.18	97.2 ± 0.8	68.3 ± 0.9
6	FeCl_3_/–/H_2_O	6.8 ± 0.2	1.50 ± 0.08	5.5 ± 0.8	1.79 ± 0.14	7.4 ± 0.3	33.1 ± 1.6
7	FeCl_3_/H_2_O_2_/H_2_O	10.4 ± 0.7	2.30 ± 0.09	7.8 ± 0.5	2.54 ± 0.04	10.6 ± 0.7	8.2 ± 1.0
8	FeCl_3_/H_2_O_2_/DMSO	15.6 ± 0.6	3.35 ± 0.15	9.7 ± 0.4	3.17 ± 0.11	20.7 ± 0.5	47.3 ± 0.6

Reaction conditions: corncob (0.200 g), FeCl_3_ (7.5 × 10^−3^ mmol), H_2_O_2_ (0.26 mL, 35 wt% in H_2_O), solvent: 2 mL, temperature: 130 °C, time: 30 min, in a 40 mL Pyrex tube with a Teflon screw cap

For a short reaction time (30 min), at 130 °C and with low FeCl_3_ concentration (3.3 mmol/L), 92% of xylose was obtained after pretreatment (Table 1, entry 3). When hydrogen peroxide was removed from the system, xylose yields dropped from 92 to 5% (Table 1, entries 3 and 2). When FeCl_3_ was removed, xylose yields dropped to 6% (Table 1, entry 1). These observations indicate that both FeCl_3_ and hydrogen peroxides are essential and have a synergistic effect on the pretreatment. This synergistic effect is due to the Fenton reaction in which the Fe cation induces the decomposition of hydrogen peroxide to produce the HO**·** (hydroxyl) and HOO**·** (perhydroxyl) radicals. These free radicals destructed the ether bond in cellulose and hemicellulose to produce xylose, glucose, arabinose, and soluble glucose oligomers with the lignin mostly intact under the pretreatment condition. In the traditional Fenton pretreatment and its combination of other pretreatment methods, a substantial amount of lignin (up to 60%) and cellulose (up to 65% of glucan) [18] were lost although the pretreated biomass had a higher cellulose/lignin ratio after pretreatment [16]. In our case, the destruction of cellulose and hemicellulose was extensive, such that almost 94% of carbohydrate in the corncob was dissolved into the pretreatment solution as monosaccharides and glucose oligomers. In addition, lignin was not extensively destructed in the pretreatment and was recovered as solid after filtration (87% recovery). These observations indicate that the HO**·** (hydroxyl) and HOO**·** (perhydroxyl) radicals generated in our pretreatment have a high selectivity towards the destruction of hemicellulose and cellulose in the lignocellulosic structure of corncob. The reason for this divergence was due to the effect of mixed solvent used in our pretreatment.

### Evaluation of solvent influence

To evaluate the effect of DMSO on the pretreatment process, pure water and pure DMSO were used as solvents under similar pretreatment conditions (Table 1, entries 7 and 8). The yields of xylose dropped from 94 to 10% when treated with pure water and dropped to 15% when treated with pure DMSO, indicating that water and DMSO have synergistic effect in the pretreatment process.

Increasing the DMSO concentration from 14.2 vol% to 25 vol% and 75 vol% in the pretreatment reduced the xylose yield from 92 to 85% and 66% (Table 1, entries 5 and 4), respectively. These results indicate that the DMSO/water ratio in the mixed solvent has an empirical ratio in order to maximize the efficiency of the pretreatment. In the organosolv pretreatment, the mixed solvent (organic solvent/water) can penetrate more effectively into the structure of biomass than pure water or organic solvent alone [46]. Our DMSO/water mixed solvent may have similar penetration ability, such that Fe cation and hydrogen peroxide, along with the HO**·** and HOO**·** radicals generated from the Fenton reaction, can enter the corncob structure more easily through the penetration of the DMSO/water solution. Replacing mixed solvent with pure water or DMSO in the pretreatment resulted in poor pretreatment efficiency, supporting the above argument. Our pretreatment temperature is slightly lower than the usual organosolv pretreatment temperature [8]. In addition, in our pretreatment, only 14.2 vol% of DMSO is required which is much lower than the organic solvent content in organosolv pretreatments (require 50% or up organic solvent) [8].

### Evaluation of hydrogen peroxide in the pretreatment

Hydrogen peroxide can remove lignin and hemicellulose from biomass, because the hydroxy-free radical produced weakens the bonding between lignin and hemicellulose [47]. Adding FeCl_3_ in hydrogen peroxide solution to promote the production of hydroxy-free radical through Fenton reaction [10] may cause the synergistic effect of FeCl_3_ and hydrogen peroxide observed in our pretreatment. Increasing the amount of hydrogen peroxide may enhance the production of hydroxyl-free radical, and thus the pretreatment efficiency. However, under such conditions, glucose can be oxidized to gluconic acid [48]. To evaluate these factors, the pretreatment process was carried out under different H_2_O_2_ concentrations. The yields of monosaccharides remained steady when the amount of H_2_O_2_ changed from 3.0 to 9.0 mmol (Table 2, entries 1 to 3), indicating that the hydrogen peroxide to FeCl_3_ concentration ratio did not alter pretreatment results in the experiment range. In addition, gluconic acid was not detected under these pretreatment conditions (Additional file 2). To confirm the absence of gluconic acid in the pretreatment, glucose was treated with the pretreatment solution for 5 h under the pretreatment condition, and gluconic acid was not detected (Additional file 3). These observations indicate that glucose cannot be oxidized to gluconic acid by the pretreatment reagent under the pretreatment conditions. The results are shown in Table 2.

**Table 2:** Effects of hydrogen peroxide and amount of corncob on the first-round pretreatment

Entry	Metal salt	Corncob (g)	H_2_O_2_ (mmol)	Xylose yield (%)	Xylose (g/L)	Glucose yield (%)	Glucose (g/L)	Arabinose yield (%)	Total carbohydrate yield (%)
1	FeCl_3_	0.2	3.0	92.2 ± 1.8	20.40 ± 0.68	20.1 ± 0.8	6.55 ± 0.07	98.4 ± 1.1	74.4 ± 1.1
2	FeCl_3_	0.2	4.5	90.4 ± 1.1	20.05 ± 0.37	22.6 ± 0.4	7.38 ± 0.31	97.3 ± 0.9	76.5 ± 1.3
3	FeCl_3_	0.2	9.0	87.3 ± 0.8	19.37 ± 0.47	16.4 ± 0.4	5.36 ± 0.54	90.5 ± 0.7	74.2 ± 0.9
4	FeCl_3_	0.4	3.0	80.7 ± 0.7	35.81 ± 0.47	19.1 ± 0.3	12.50 ± 0.42	82.4 ± 0.7	70.1 ± 1.1
5	FeCl_3_	0.6	3.0	77. 4 ± 1.1	51.52 ± 0.73	17.5 ± 0.6	17.17 ± 0.43	88.1 ± 0.5	70.8 ± 0.6
6	FeCl_3_	0.8	3.0	64.7 ± 1.3	57.43 ± 1.13	18.6 ± 0.4	24.32 ± 0.65	82.7 ± 0.6	61.5 ± 0.9

Reaction conditions: Corncob, FeCl_3_ (7.5 × 10^−3^ mmol, 3.3 mmol/L), H_2_O_2_ (35 wt% in H_2_O), solvent: 2 mL (DMSO/water, 1:6), temperature: 130 °C, time: 30 min, in a 40 mL Pyrex tube with a Teflon screw cap

### Evaluation of pretreatment temperature and the pretreatment time

**Fig. 2:**
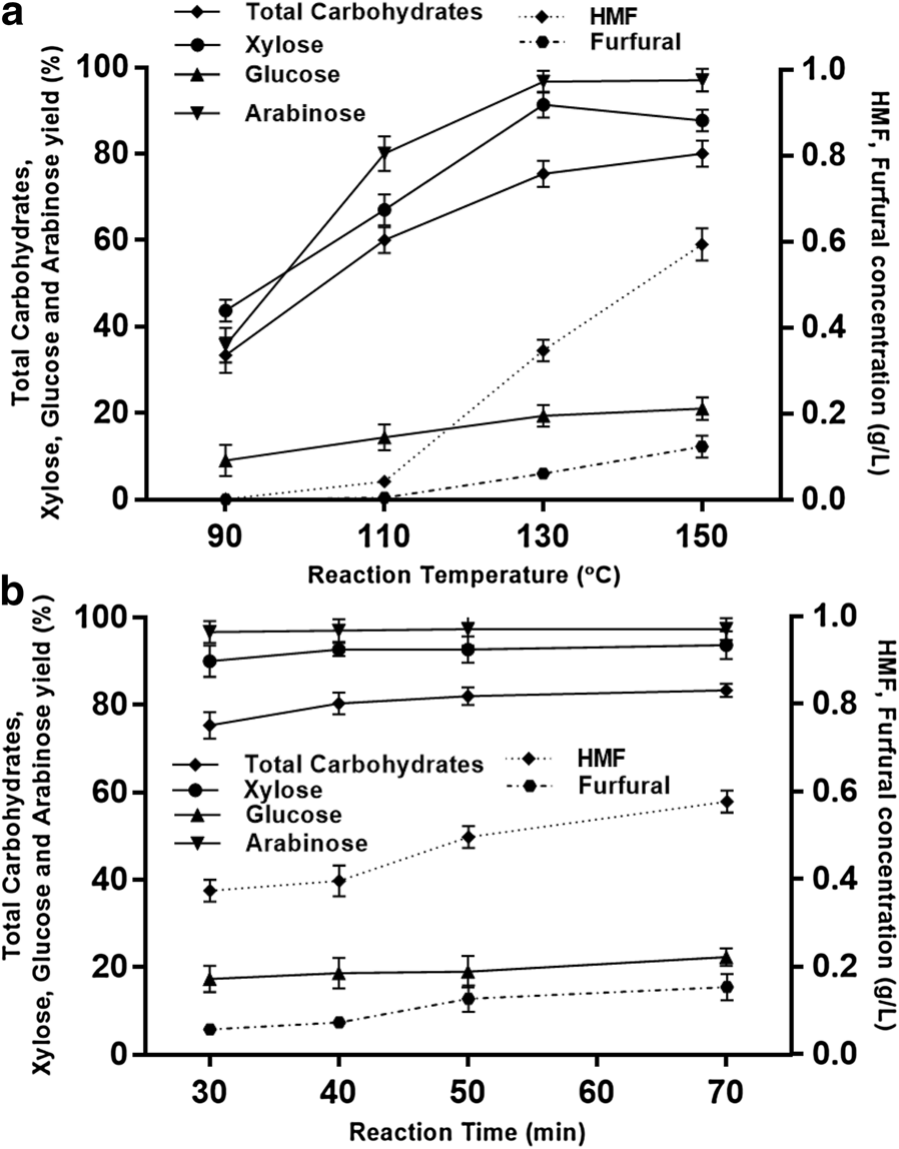
Effects of **a** temperature on the pretreatment of 0.200 g corncob using FeCl_3_ (7.5 × 10^−3^ mmol) with H_2_O_2_ (35 wt% in H_2_O, 3.0 mmol, 0.260 mL) in DMSO/water (1:6, 2.0 mL) for 30 min and **b** reaction time on the pretreatment of 0.200 g corncob using FeCl_3_ (7.5 × 10^−3^ mmol) with H_2_O_2_ (35 wt% in H_2_O, 3.0 mmol, 0.260 mL) in DMSO/water (1:6, 2.0 mL) at 130 °C

Based on the results of H_2_O_2_ evaluation mentioned above, we used 3.0 mmol of hydrogen peroxide for the evaluation of pretreatment temperature. The results of influence of pretreatment temperature on the monosaccharides yield are shown in Fig. 2a.

Generally speaking, increasing pretreatment temperature enhances the monosaccharides yield. However, at higher temperature, the amount of HMF and furfural, inhibitors of enzymatic hydrolysis, also increased [49]. The higher amount of HMF and furfural may be due to the higher conversion of glucose to HMF and furfural at higher temperature (Additional file 4: Table S3; Additional file 5). To optimize the pretreatment efficiency (higher carbohydrate recovery and higher yield for monosaccharides) and considering the subsequent enzymatic hydrolysis (less inhibitor is favored), pretreatment temperature was set at 130 °C. Both xylose and glucose yields remained high from 30 to 70 min. Longer pretreatment time did not increase the xylose and glucose yield significantly. However, for longer pretreatment time, the amount of inhibitors, furfural and HMF, increased 20% and 50%, respectively (Additional file 6: Table S4). Gluconic acid was not detected in all these pretreatment conditions (Additional files 4 and 6: Table S3 and S4). These results are shown in Fig. 2B.

### Optimization of biomass amount

Based on our evaluations of the influence of FeCl_3_, solvent effect hydrogen peroxide, pretreatment temperature, and time on pretreatment outcomes, the optimized pretreatment conditions for corncob were set as follows: FeCl_3_ (7.5 × 10^−3^ mmol, 3.3 mmol/L), H_2_O_2_ (0.26 mL, 35 wt% in H_2_O), solvent: 2 mL (DMSO/water), temperature: 130 °C, and time: 30 min. These conditions are carried out in a 40 mL Pyrex tube with a Teflon screw cap.

To determine the maximum amount of corncob that can be used in each pretreatment, we tested a wide range of corncob input while maintaining same pretreatment conditions. The results are shown in Table 2, entries 4–6. We show that the amount of corncob in each pretreatment can be increased threefold to 0.6 g/2.26 mL (corresponding to 265.5 g/L). The concentration of monosaccharides of the resulting filtrate for the first round of pretreatment are 17.2 g/L for glucose, 51.5 g/L for xylose, and 54.2 g/L for glucose oligomers and other sugar oligomers.

### Enzymatic hydrolysis of glucose oligomers in the pretreatment filtrate

**Fig. 3:**
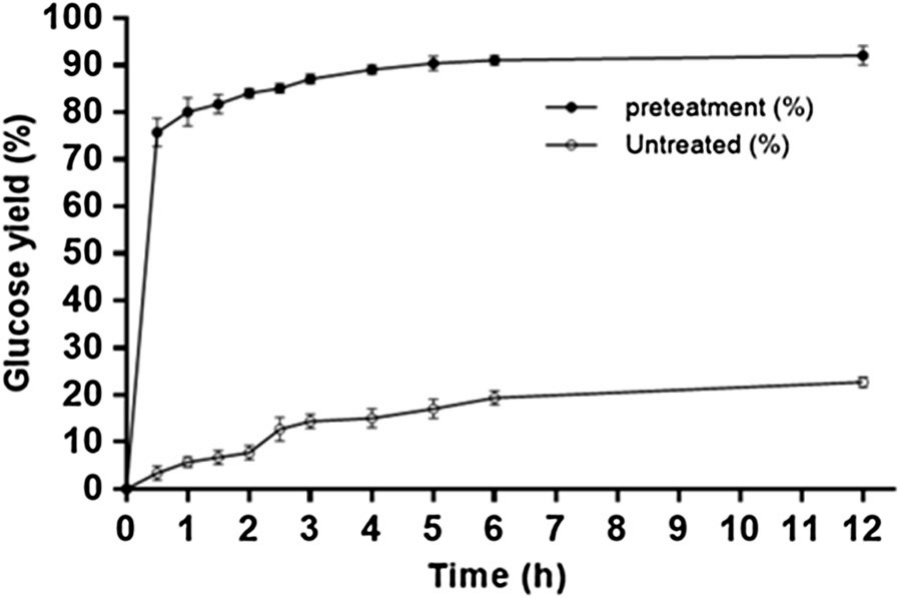
Time course of total glucose yields of the enzymatic hydrolysis of the pretreated (filtrate from pretreatment) and the untreated (corncob powder) feedstocks

The total filtrate (from both first- and second-round pretreatment of 0.200 g corncob) contained 0.124 g carbohydrates within which 0.076 g was xylose (0.046 g) and glucose (0.030 g). The other 0.041 g carbohydrate was glucose oligomers and other sugar oligomers (Fig. 1). The filtrate was applied to enzymatic hydrolysis to convert the soluble glucose oligomers to glucose. The row corncob was applied to hydrolysis under the same conditions for comparison. Cellulase from *Trichoderma reesei* was used and the results are shown in Fig. 3.

After 12 h, 0.068 g glucose corresponding to 92% of the row corncob glucose was obtained. Considering the original amount of glucose (0.030 g) in the filtrate before hydrolysis, 0.038 g glucose was obtained through the enzyme hydrolysis of the glucose oligomers in the filtrate. This hydrolysate solution has a glucose concentration of 15.0 g/L and can be directly used as a feedstock for the subsequent fermentation to produce ethanol. For the hydrolysis of row corncob, only 21 wt% of its glucose content was obtained in the hydrolysis.

**Fig. 4:**
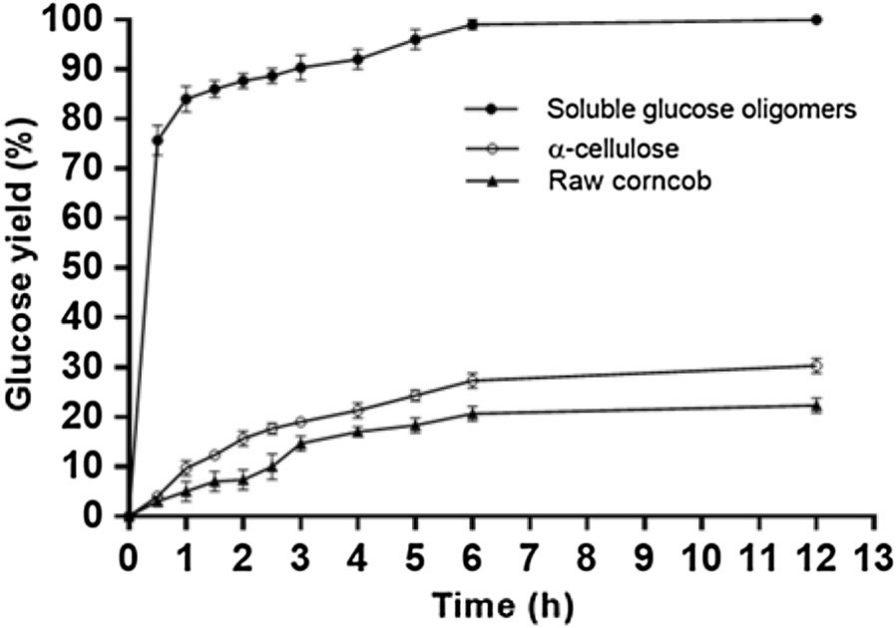
Time course of glucose yield of the enzymatic hydrolysis of the filtrate containing 0.038 g soluble glucose oligomers, α-cellulose (0.038 g) and raw corncob (0.103 g) with similar glucose content (0.038 g glucose)

To show the glucose oligomer in the filtrate which could be hydrolysed more effectively than crystalline cellulose and the cellulose in the raw corncob, corresponding amount of commercial cellulose (0.038 g) and corncob (0.103 g) containing the same amount of glucose as the soluble glucose oligomers (0.038 g) in the filtrate were applied to enzymatic hydrolysis for comparison. The time course of enzymatic hydrolysis results is summarized in Fig. 4.

After 6 h, 98 wt% of the glucose oligomers in the filtrate was hydrolyzed to glucose, and only 30 wt% of corresponding amount of commercial cellulose was hydrolyzed to glucose under similar condition. For the raw corncob, 20 wt% of its glucose content was hydrolysed to glucose. These observations indicate that the filtrate which contains FeCl_3_, DMSO, and trace amount of inhibitors (HMF and furfural) can be applied to enzymatic hydrolysis. The higher efficiency of the glucose oligomers hydrolysis is understandable, because soluble glucose oligomers obtained after the pretreatment have a higher accessibility towards cellulase than that of crystalline cellulose and the cellulose in the raw corncob which is protected by the lignin and hemicellulose [50].

### Fermentability of the pretreatment hydrolysate

**Fig. 5:**
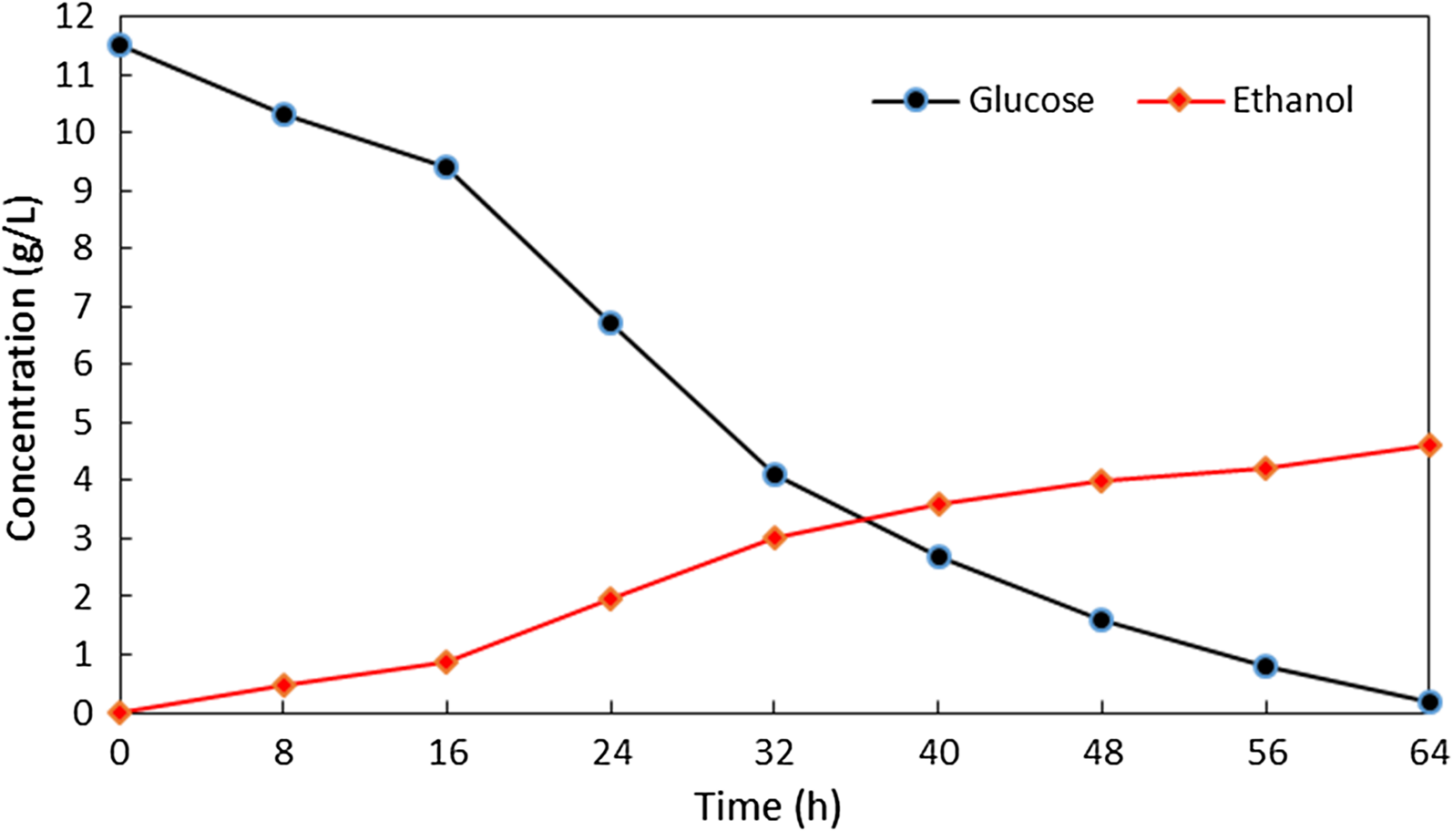
Time courses for the ethanol fermentation of hydrolyzate containing glucose

The hydrolysate obtained in the enzymatic hydrolysis of the pretreatment filtrate was directly applied to ethanol fermentation using *S. cerevisiae* [15]. The time courses for the ethanol fermentation of hydrolysate from the enzymatic hydrolysis of the pretreatment filtrate containing 11.5 g/L glucose and 8.3 g/L xylose is shown in Fig. 5.

After 24 h, the concentration of glucose reduced to 60% (from 11.5 to 6.7 g/L), and the concentration of ethanol was 1.96 g/L. After 64 h, the concentrations of ethanol and glucose reached 4.6 g/L and 0.2 g/L, respectively. The ethanol yield was 0.41 g (ethanol)/g (glucose) which equals to 79% of theoretical yield. These results indicate that the DMSO and FeCl_3_ in the hydrolysate have a little effect in the fermentation of glucose to ethanol.

### Pretreatment by iron oxide

Because FeCl_3_ can be oxidized to iron oxide in aqueous solution [51], iron oxide may form during the pretreatment. It would be interesting to evaluate whether iron oxide can replace FeCl_3_ in the pretreatment (Additional file 7: Scheme S1) [52].

When iron oxide was used, the total carbohydrates obtained in the pretreatment filtrate were 0.082 g (62% of total carbohydrates in the corncob) with 0.040 g of xylose corresponding to 80% xylose of the corncob. The residue obtained in the first round of pretreatment was applied to subsequent rounds of pretreatment for totally 8 h, and 0.048 g of carbohydrates corresponding to 36.3% of carbohydrates in the corncob was obtained. The total carbohydrates obtained in the filtrates are 0.130 g corresponding to 98.2% of total carbohydrates in the corncob which was recovered. The efficiency of pretreatment using Fe_3_O_4_ is comparable to that of using FeCl_3_. However, Fe_3_O_4_ can be removed from the filtrate more easily and effectively by filtration.

There are several advantages of using iron oxide in the process. First, it is environmental benign. Second, it can be recovered in the filtration step. Third, it can be removed from residues by magnetic, because Fe_3_O_4_ is ferrimagnetic.

## Conclusions

A modified Fenton pretreatment of corncob using low concentration of FeCl_3_ (3.3 mmol/L) and hydrogen peroxide in mixed solvent (DMSO/water) at 130 °C was developed. The pretreatment process is simple and efficient with 94% recovery of carbohydrates as soluble monosaccharide (92% xylose and 40% of glucose) and glucose oligomers in the filtrate. Such filtrate was directly applied to the subsequent enzymatic hydrolysis and where 92% of the corncob glucose content was obtained. The hydrolysate so obtained was directly applied to ethanol fermentation with good fermentability. This pretreatment condition is mild (130 °C, 1.5 bar), and the additives and solvents used in this pretreatment method have a low impact to the environment. We also show that, in this method, FeCl_3_ can be replaced by ferromagnetic Fe_3_O_4_ with slightly lower efficiency. This method now provides the opportunity to substantially maximize the carbohydrate and solid lignin recovery of biomass with a comparatively green process, such that the efficiency of biorefinery as well as the bioethanol production process can be improved. The present pretreatment is still relatively energy intensive and expensive (drying, grinding, comparatively expensive organic solvent, etc.), and further optimization of the process, such as open sun drying and with less grinding (bigger particular size) may be required in large-scale operation.

## Additional files

**Additional file 1: Figure S1.** Spectra for (a) TGA and (b) DTG of raw and pretreated Corncob.

**Additional file 2.** Quantitative analysis of glucose and gluconic acid by LC–MS on the corncob pretreatment filtrate.

**Additional file 3.** Quantitative analysis of gluconic acid and glucose by LC–MS on pretreatment reagent treated glucose.

**Additional file 4: Table S3.** Amount of inhibitors produced at different temperatures.

**Additional file 5.** Amount of HMF and furfural produced in the corncob pretreatments under different conditions.

**Additional file 6: Table S4.** Amount of inhibitors produced in different reaction time.

**Additional file 7: Scheme S1.** Corncob pretreatment using Fe_3_O_4_.
