# Supplementary material for: Efficient pretreatment of lignocellulosic biomass with high recovery of solid lignin and fermentable sugars using Fenton reaction in a mixed solvent
Source: Biotechnol Biofuels. 2018 Oct 20;11:287. doi: 10.1186/s13068-018-1288-4 (PMC6195684; doi:10.1186/s13068-018-1288-4)
Supplement: Supplementary file 1 — Additional file 1: Figure S1. Spectra for (a) TGA and (b) DTG of raw and pretreated Corncob. [file 13068_2018_1288_MOESM1_ESM.docx]

Additional file 1

**TGA**

**
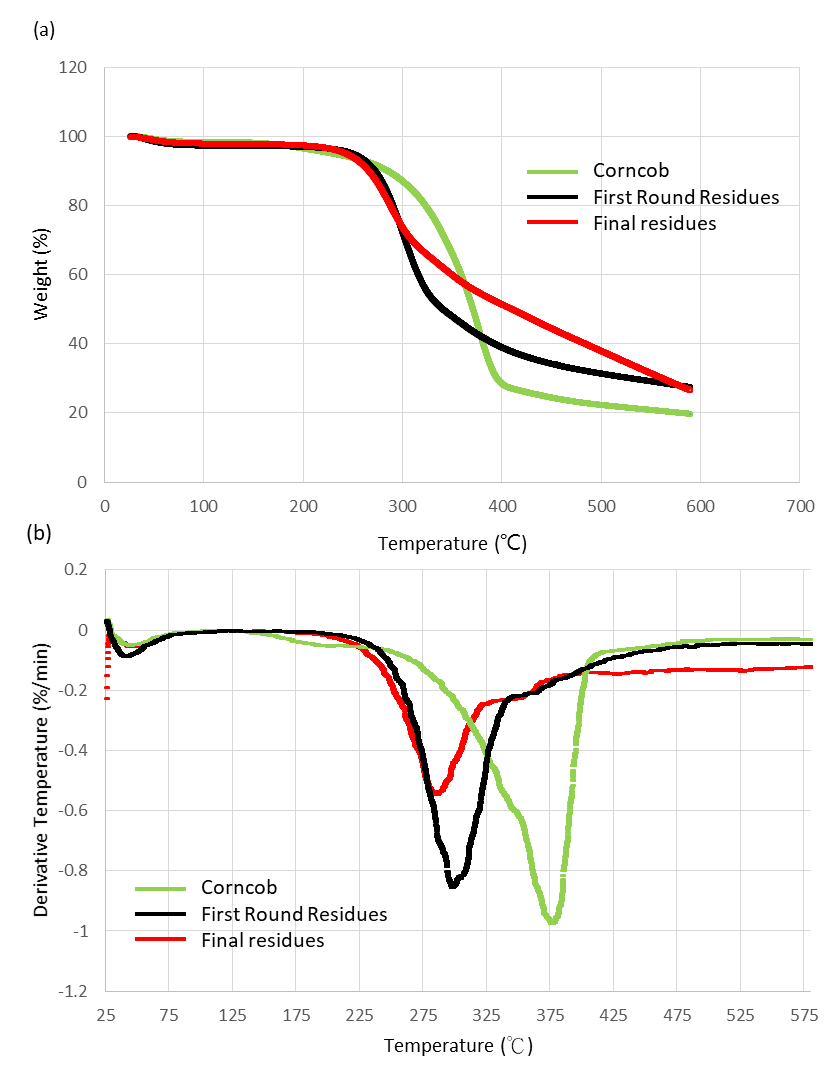
**

Fig. S1 Spectra for (a) TGA and (b) DTG of raw and pretreated Corncob

The pyrolysis experiments were performed in a sensitive thermobalance (Perkin-Elmer, Pyris1 TGA) at a heating rate of 10 ℃/min up to a final temperature of 600 ℃ under the helium flow rate of 50 ml/min.
