# Supplementary material for: Efficient pretreatment of lignocellulosic biomass with high recovery of solid lignin and fermentable sugars using Fenton reaction in a mixed solvent
Source: Biotechnol Biofuels. 2018 Oct 20;11:287. doi: 10.1186/s13068-018-1288-4 (PMC6195684; doi:10.1186/s13068-018-1288-4)
Supplement: Supplementary file 2 — Additional file 2. Quantitative analysis of glucose and gluconic acid by LC–MS on the corncob pretreatment filtrate. [file 13068_2018_1288_MOESM2_ESM.docx]

Additional file 2

Quantitative analysis of glucose and gluconic acid by LC-MS on the corncob pretreatment filtrate.

Table S1 Amount of gluconic acid in the pretreatment filtrate.^a^

| Entry | Feedstock | FeCl_3_/H_2_O_2_/Solvent | Glucose Yield (%) | Gluconic acid Yield (%) |
| --- | --- | --- | --- | --- |
| 1 | Corncob | FeCl_3_ / H_2_O_2_ (3 mmol)/  DMSO + H_2_O (1:6) | 6.55 ± 0.07 | N.D.^b^ |
| 2 | Corncob | FeCl_3_ / H_2_O_2_ (4.5 mmol)/  DMSO + H_2_O (1:6) | 7.38 ± 0.31 | N.D. |
| 3 | Corncob | FeCl_3_ / H_2_O_2_ (9 mmol)/  DMSO + H_2_O (1:6) | 5.36 ± 0.54 | N.D. |
| ^a^ Reaction Conditions: Corncob (0.200 g), FeCl_3_ (7.5x10^-3^ mmol, 3.3 mmol/L), H_2_O_2_ (35 wt% in H_2_O), solvent: 2 mL, temperature: 130 ℃, time: 30 min, in a 40 mL Pyrex tube with a Teflon screw cap.  ^b^ Not detected. | | | | |

Chromatographic separations were performed on a Merck ZIC-HILIC column (2.1 x 150 mm, 3.5 um) using mobile phase A (acetonitrile modified with 0.1% (v/v) formic acid) and mobile phase B (5.0 mmol/L ammonium acetate modified with 0.1% (v/v) formic acid) with gradient profile 10% B to 90% B in 19 min and followed by 6 min of column washing with a linear gradient from 90% to 10% B including column re-equilibration with 10% B for glucose and gluconic acid. Analyses were performed at a flow rate of 0.30 mL/min. Data acquisition was observed in multiple resonances monitoring (MRM) mode by using ESI-MS/MS instrument. Ion monitored for glucose, precursor monitored were m/z 225 and fragment ion were m/z 118.7 in negative mode; for gluconic acid, precursor monitored were m/z 195 and fragment ion were m/z 75.3 in negative mode.

|  | **M’** | **[M+COOH]^-^** | **Fragment** | **R. T.(min)** |
| --- | --- | --- | --- | --- |
| Glucose | M=180 | 225 | 118.7 | 4.26 |
|  | **M’** | **[M-H]-** | **Fragment** | **R. T.(min)** |
| Gluconic Acid | M=196 | 195 | 75.3 | 5.25 |


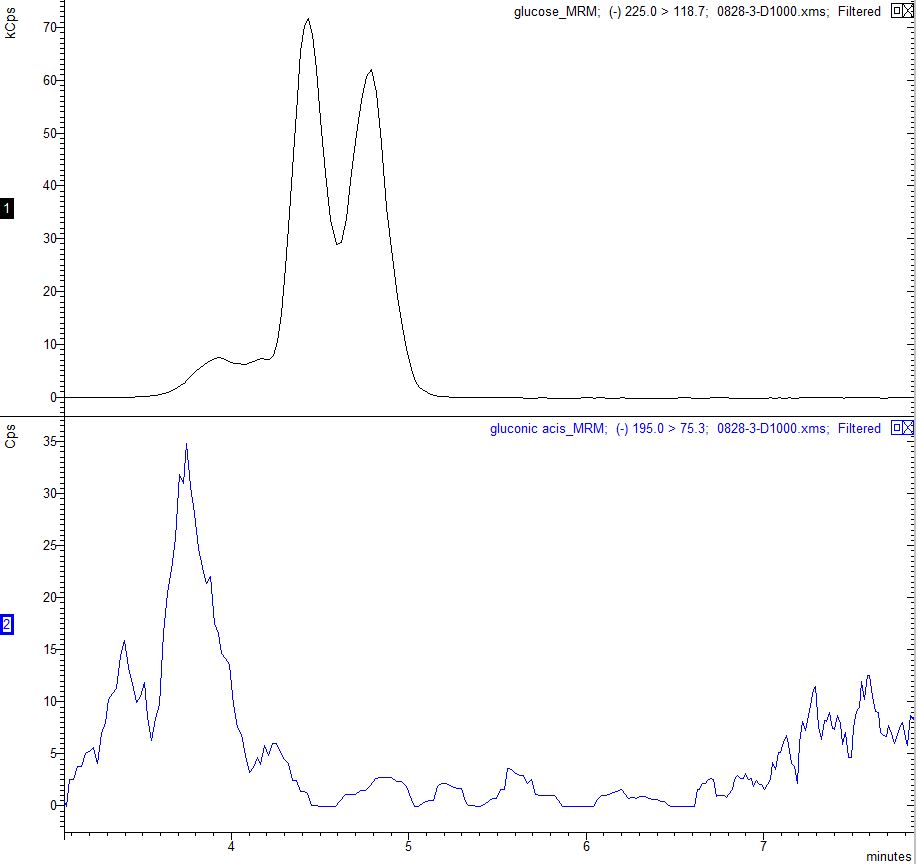


Fig S2 LC-MS spectra of glucose and gluconic acid of the pretreatment filtrate observed in MRM mode by using ESI-MS/MS.


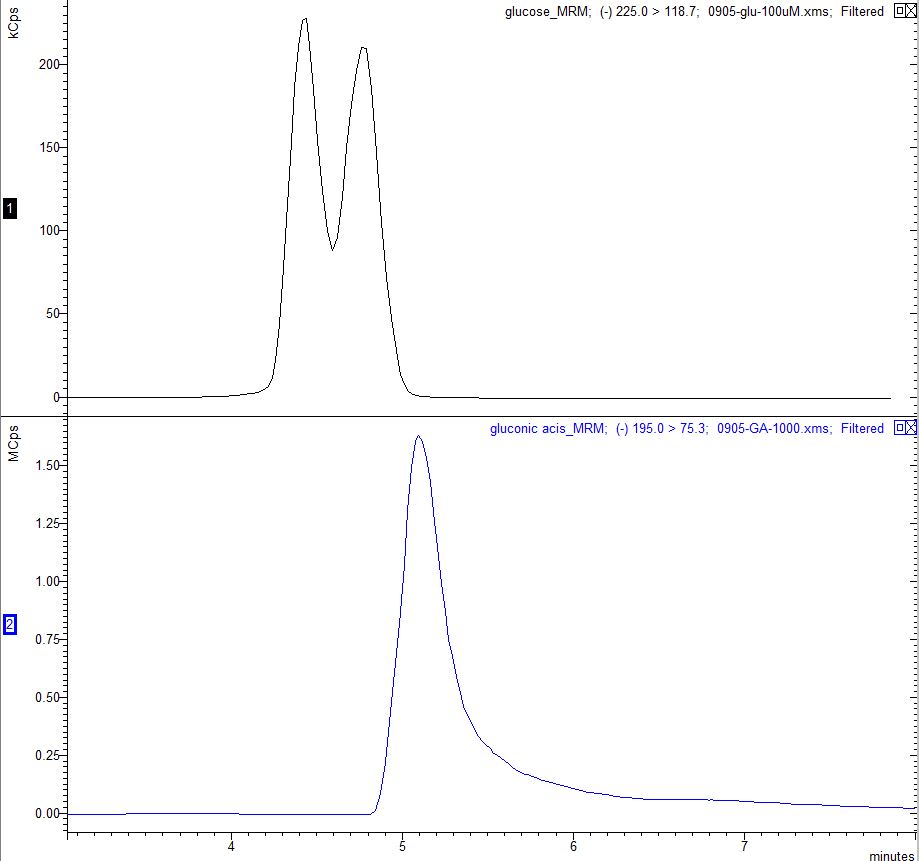


Fig S3 LC-MS spectra of glucose and gluconic acid of glucose/gluconic acid (1:10) solution observed in MRM mode by using ESI-MS/MS.
