# Supplementary material for: Efficient pretreatment of lignocellulosic biomass with high recovery of solid lignin and fermentable sugars using Fenton reaction in a mixed solvent
Source: Biotechnol Biofuels. 2018 Oct 20;11:287. doi: 10.1186/s13068-018-1288-4 (PMC6195684; doi:10.1186/s13068-018-1288-4)
Supplement: Supplementary file 5 — Additional file 5. Amount of HMF and furfural produced in the corncob pretreatments under different conditions. [file 13068_2018_1288_MOESM5_ESM.docx]

Additional file 5

**Amount of HMF and furfural produced in the corncob pretreatment under different conditions.**^a^

| Entry | Corncob (g) | FeCl_3_ (mmol) | H_2_O_2_ (mmol) | Solvent | Furfural  (g/L) | HMF  (g/L) |
| --- | --- | --- | --- | --- | --- | --- |
| 1 | 0.2 | --- | 3.0 | DMSO + H_2_O (1:6) | 0.0041 ± 0.0003 | 0.033 ± 0.004 |
| 2 | 0.2 | 7.5x10^-3^ | --- | DMSO + H_2_O (1:6) | 0.0015 ± 0.0002 | 0.017 ± 0.003 |
| 3 | 0.2 | 7.5x10^-3^ | 3.0 | DMSO + H_2_O (1:6) | 0.0633 ± 0.0051 | 0.347 ± 0.025 |
| 4 | 0.2 | 7.5x10^-3^ | 3.0 | DMSO + H_2_O (3:1) | 0.3683 ± 0.0038 | 2.525 ± 0.051 |
| 5 | 0.2 | 7.5x10^-3^ | 3.0 | DMSO + H_2_O (1:3) | 0.1023 ± 0.0331 | 0.703 ± 0.021 |
| 6 | 0.2 | 7.5x10^-3^ | --- | H_2_O | 0.0011 ± 0.0002 | 0.013 ± 0.005 |
| 7 | 0.2 | 7.5x10^-3^ | 3.0 | H_2_O | 0.0161 ± 0.0032 | 0.153 ± 0.031 |
| 8 | 0.2 | 7.5x10^-3^ | 3.0 | DMSO | 3.2266 ± 0.0271 | 12.110 ± 0.083 |
| 9 | 0.2 | 7.5x10^-3^ | 4.5 | DMSO + H_2_O (1:6) | 0.0751 ± 0.0024 | 0.319 ± 0.033 |
| 10 | 0.2 | 7.5x10^-3^ | 9.0 | DMSO + H_2_O (1:6) | 0.0773 ± 0.0045 | 0.543 ± 0.024 |
| 11 | 0.4 | 7.5x10^-3^ | 3.0 | DMSO + H_2_O (1:6) | 0.1134 ± 0.0213 | 0.513 ± 0.037 |
| 12 | 0.6 | 7.5x10^-3^ | 3.0 | DMSO + H_2_O (1:6) | 0.1352 ± 0.0352 | 0.811 ± 0.013 |
| 13 | 0.8 | 7.5x10^-3^ | 3.0 | DMSO + H_2_O (1:6) | 0.1462 ± 0.0147 | - 1. ± 0.041 |
| ^a^ Reaction Conditions: Corncob, FeCl_3_ , H_2_O_2_ (35 wt% in H_2_O), solvent: 2 mL, temperature: 130 ℃, time: 30 min, in a 40 mL Pyrex tube with a Teflon screw cap. | | | | | | |
