# Supplementary material for: Efficient pretreatment of lignocellulosic biomass with high recovery of solid lignin and fermentable sugars using Fenton reaction in a mixed solvent
Source: Biotechnol Biofuels. 2018 Oct 20;11:287. doi: 10.1186/s13068-018-1288-4 (PMC6195684; doi:10.1186/s13068-018-1288-4)
Supplement: Supplementary file 6 — Additional file 6: Table S4. Amount of inhibitors produced in different reaction time. [file 13068_2018_1288_MOESM6_ESM.docx]

Additional file 6

Table S4 Amount of inhibitors produced in different reaction time.^a^

| Pretreatment time | Furfural  (g/L) | HMF  (g/L) | Gluconic acid  (g/L) |
| --- | --- | --- | --- |
| 30 min | 0.065 ± 0.004 | 0.373 ± 0.025 | N.D.^b^ |
| 40 min | 0.074 ± 0.004 | 0.397 ± 0.035 | N.D. |
| 50 min | 0.120 ±0.020 | 0.427 ± 0.020 | N.D. |
| 70 min | 0.160 ± 0.023 | 0.550 ± 0.031 | N.D. |

^a^ Reaction Conditions: Corncob (0.2 g), metal salt: FeCl_3_ (7.5x10^-3^ mmol), additive: H_2_O_2_ (0.26 mL, 35 wt% in H_2_O), solvent: 2 mL (Dimethyl Sulfoxide/water, 1:6), temperature: 130 ℃, in a 40 mL Pyrex tube with a Teflon screw cap

^b^ Not detected.

Chromatographic separations were performed on a Waters Acquity UPLC BEH C18 column (2.1 x 100 mm, 1.7 um) using an isocratic mixture of 0.01 mmol/L acetic acid in 0.2% aqueous solution of formic acid for HMF compound; and Merck ZIC-HILIC column (2.1 x 150 mm, 3.5 um) using mobile phase A (acetonitrile modified with 0.1% (v/v) formic acid) and mobile phase B (5.0 mmol/L ammonium acetate modified with 0.1% (v/v) formic acid) with gradient profile 10% B to 90% B in 19 min for gluconic acid. Both analyses were performed at a flow rate of 0.30 mL/min. Data acquisition was observed in multiple resonances monitoring (MRM) mode. Ion monitored for HMF were m/z 127.0 and fragment ion were m/z 59.4 and the precursor for furfural were m/z 97.0 and fragment ion were m/z 118.7 in positive mode; for gluconic acid, precursor monitored were m/z 195 and fragment ion were m/z 75.3 in negative mode.

|  | **M’** | **[M+H]^+^** | **Fragment** | **R. T.(min)** |
| --- | --- | --- | --- | --- |
| Furfural | M=96 | 97 | 118.7 | 4.26 |
| HMF | M=126 | 127 | 59.4 | 3.25 |
|  | **M’** | **[M-H]-** | **Fragment** | **R. T.(min)** |
| Gluconic Acid | M=196 | 195 | 75.3 | 5.25 |
