# Supplementary figures and images for: Efficient pretreatment of lignocellulosic biomass with high recovery of solid lignin and fermentable sugars using Fenton reaction in a mixed solvent
Source: Biotechnol Biofuels. 2018 Oct 20;11:287. doi: 10.1186/s13068-018-1288-4 (PMC6195684; doi:10.1186/s13068-018-1288-4)

Additional file 7

**Pretreatment by iron oxide**

**
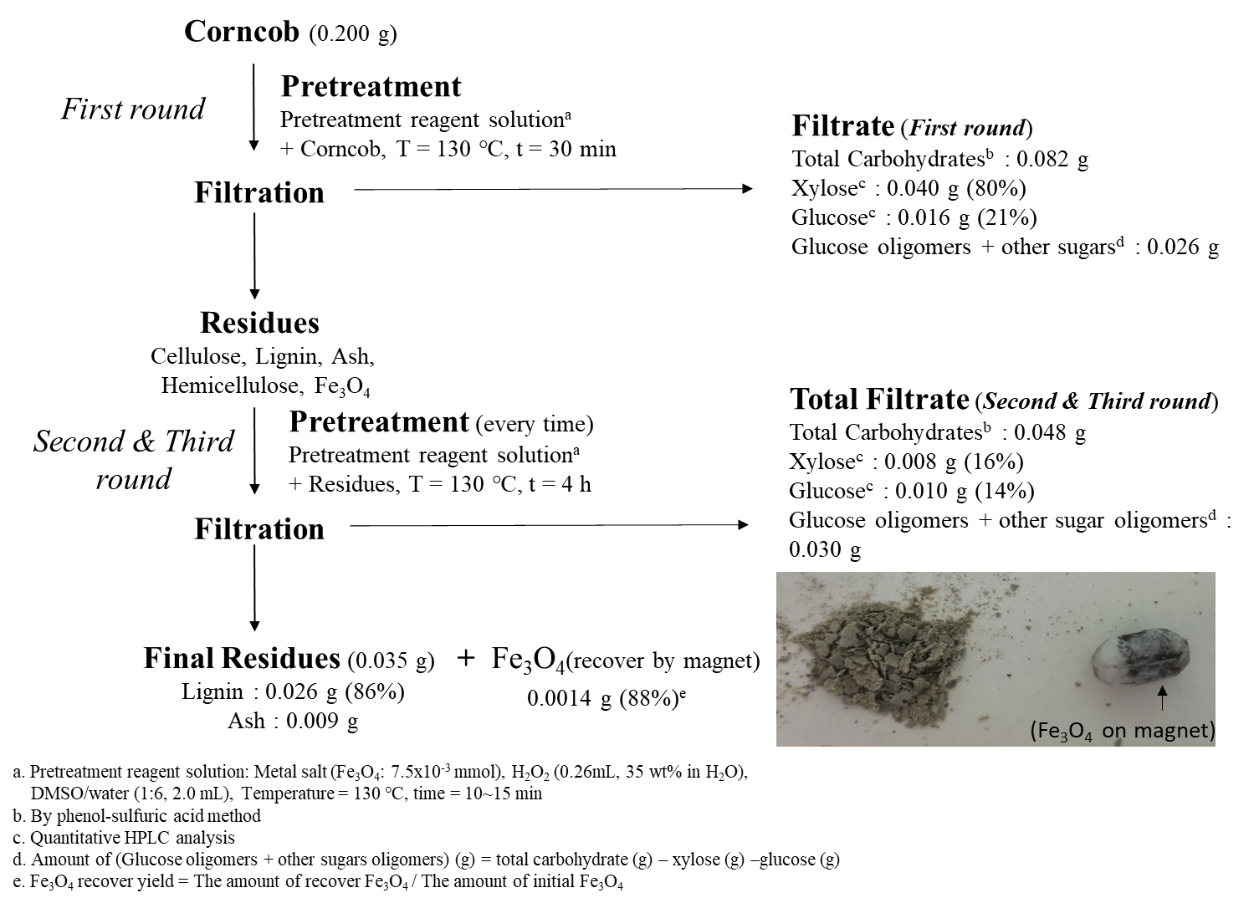
**

Scheme S1. Corncob pretreatment using Fe3O4

Supplement: Supplementary file 7 — Additional file 7: Scheme S1. Corncob pretreatment using Fe3O4. [file 13068_2018_1288_MOESM7_ESM.docx]
